# A new genus of Gnorimoschemini (Lepidoptera, Gelechiidae, Gelechiinae) from the Atacama and Sechura deserts

**DOI:** 10.3897/zookeys.1285.182943

**Published:** 2026-07-16

**Authors:** Jimena Ampuero-Vega, Ricardo Campos-Soto, Hector A. Vargas

**Affiliations:** 1 Departamento de Negocios, Facultad de Ingeniería, Negocios y Ciencias Ambientales, Universidad Viña del Mar, Viña del Mar, Chile Departamento de Negocios, Facultad de Ingeniería, Negocios y Ciencias Ambientales, Universidad Viña del Mar Viña del Mar Chile; 2 Research Institute CIBIO (Centro Iberoamericano de la Biodiversidad), Scientific Park, University of Alicante, Ctra. San Vicente del Raspeig s/n, E-03690 San Vicente del Raspeig, Alicante, Spain Departamento de Ciencias Veterinarias, Facultad de Ciencias de la Vida, Universidad Viña del Mar Viña del Mar Chile; 3 Departamento de Ciencias Veterinarias, Facultad de Ciencias de la Vida, Universidad Viña del Mar, Viña del Mar, Chile Departamento de Recursos Ambientales, Facultad de Ciencias Agronómicas, Universidad de Tarapacá Arica Chile; 4 Departamento de Recursos Ambientales, Facultad de Ciencias Agronómicas, Universidad de Tarapacá, Arica, Chile Research Institute CIBIO (Centro Iberoamericano de la Biodiversidad), Scientific Park, University of Alicante Alicante Spain

**Keywords:** Asteraceae, DNA barcoding, leaf miners, moths, new combination, new species, sexual dimorphism

## Abstract

Sexually dimorphic Gnorimoschemini moths (Lepidoptera, Gelechiidae, Gelechiinae) were reared from leaf mines collected on *Tessaria absinthioides* (Hook. & Arn.) DC. (Asteraceae) in the coastal Atacama Desert of northern Chile. Detailed examination revealed a combination of morphological characters not assignable to any known genus of the tribe, including 1) a scale tuft on the male hindwing, 2) an uncus with a triangular postero-medial projection, and 3) a U-shaped gnathos. COI sequences obtained from a male and a female showed minimal intraspecific divergence (0.6%), whereas interspecific divergences with representatives of other Gnorimoschemini genera ranged from 8.1% to 11.8%. Phylogenetic analyses based on COI sequences further confirmed the Atacama moths as a strongly supported, isolated clade, corroborating its morphological distinctiveness and supporting its recognition as a separate evolutionary lineage. The combined evidence justifies the establishment of *Azaptilia azapensis* Vargas, **gen. nov. et sp. nov**. Furthermore, based on the remarkable similarity in genitalia morphology, the Peruvian moth *Scrobipalpula trichinaspis* (Meyrick, 1917) from the Sechura Desert, originally described in *Phthorimaea* Meyrick, 1902, is also included in the new genus as *Azaptilia trichinaspis* (Meyrick, 1917), **comb. nov**. This discovery expands the known diversity of Gnorimoschemini in arid South American ecosystems of the Atacama and Sechura deserts and highlights the importance of integrating rearing, morphology, and DNA barcoding for uncovering hidden diversity in Neotropical Gelechiidae.

## Introduction

The widespread tribe Gnorimoschemini (Lepidoptera, Gelechiidae, Gelechiinae) includes 986 described species assigned to 39 genera (Hobern et al. 2025). Denser sampling and a longer tradition of taxonomic studies in the Palaearctic have resulted in more than half of the currently described species being recorded from this region (Powell and Povolný 2001; Povolný 2002). However, the tribe likely originated in xeric environments of South America, such as parts of the Andes and Patagonia (Povolný 1994; Powell and Povolný 2001). Although knowledge of Neotropical Gnorimoschemini diversity has improved substantially due to taxonomic work during the last century (Povolný 1985, 1986, 1987, 1989, 1990, 1994), recent discoveries suggest that many Neotropical lineages remain unknown and that further fieldwork is essential to uncover them (Landry and Roque-Albelo 2010; Cepeda 2019; Bidzilya and Corro Chang 2022).

Some members of Gnorimoschemini are important agricultural pests, and consequently their biology has been widely studied (Corro Chang and Metz 2021). However, the biology of many others remains poorly understood. Larvae of the tribe show a wide diversity of feeding habits, with most being concealed feeders (Powell and Povolný 2001). The few available feeding records for Neotropical representatives include leaf-mining, leaf-tying, flower-feeding, seed-feeding, gall-inducing, and gall-inquiline larvae associated with plants from at least six different families (Landry and Roque-Albelo 2010; Adamski et al. 2018; Vargas 2019, 2020, 2022, 2023, 2025).

The monophyly of Gnorimoschemini has been supported by a multilocus molecular phylogenetic analysis (Karsholt et al. 2013), whereas morphology has not provided conclusive evidence to recognize this tribe as a natural group (Nazari and Landry 2012). The distinctive male genital morphology supports monophyly in some genera (Nazari and Landry 2012), but morphological differences among others are subtle, making the identification of likely synapomorphies difficult (Povolný 1994, 2002). As a result, the validity of some genera has been controversial, and generic assignment for some species can be extremely challenging (Lee et al. 2009; Corro Chang and Metz 2021). In such cases, phylogenetic analyses based on mitochondrial DNA sequences serve as a useful complementary tool for generic assignment in different tribes of Gelechiidae (Nazari 2017; Metz et al. 2019; Corley et al. 2020).

Sexually dimorphic moths belonging to an undescribed species of Gnorimoschemini were obtained from leaf mines collected on the shrub *Tessaria absinthioides* (Hook. & Arn.) DC. (Asteraceae) in the transverse valleys of the Atacama Desert, northern Chile. Since several morphological features preclude assigning the new species to any previously described Gnorimoschemini genera, and a phylogenetic analysis based on mitochondrial DNA sequences supports the uniqueness suggested by the morphology, the lineage discovered in the Atacama Desert is described here as a new genus and species of Gnorimoschemini. In addition, *Scrobipalpula trichinaspis* (Meyrick, 1917), a Peruvian moth from the Sechura Desert, is also included in the new genus based on genitalia morphology.

## Material and methods

### Morphological observations

The moths examined in this study were obtained in December 2024 from mined leaves of *T. absinthioides* collected in October 2024 in the Azapa Valley (18°35'05"S, 69°52'19"W), at about 1050 m elevation in the Atacama Desert, Arica Province, northern Chile. The abdomen of each specimen was removed and placed in hot potassium hydroxide (KOH) 10% for a few minutes for dissection of the genitalia. The genitalia were then stained with Eosin Y and Chlorazol Black and mounted on slides with Euparal. Photos of the habitus and genitalia were taken with an iPhone 11 camera attached to a Leica M125 stereomicroscope and a Leica DM1000 LED microscope, respectively. The holotype, paratypes, and their genitalia slides are deposited in the “Colección Entomológica de la Universidad de Tarapacá” (IDEA), Arica, Chile.

### Molecular analysis

Genomic DNA was extracted from two legs of one male (holotype) and one female (paratype) using the QIAamp Fast DNA Tissue Kit, following the manufacturer’s instructions. DNA purification, PCR amplification, and sequencing of the barcode region (Hebert et al. 2003) were performed using the primers LCO1490 and HCO2198 (Folmer et al. 1994) at Macrogen Inc. (Seoul, South Korea). The PCR program included 5 min at 94 °C, 35 cycles of 30 s at 94 °C, 30 s at 47 °C, 1 min at 72 °C, and a final elongation step of 10 min at 72 °C. The chromatograms were viewed in Chromas 2.6.6 (Technelysium Pty. Ltd, Australia), and consensus sequences were generated in MEGA 11 (Tamura et al. 2021). The obtained sequences were deposited in the Barcode of Life Data System (BOLD) (Ratnasingham and Hebert 2007) under process IDs NCMIC020-25 and NCMIC021-25. To assess the generic assignment of the new species, the obtained sequences were used to calculate genetic distances and to conduct phylogenetic analyses using maximum likelihood (ML) and Bayesian inference (BI), together with sequences of other members of Gnorimoschemini downloaded from BOLD (Table 1). Due to the similarity in genitalia morphology between the new species and *S. trichinaspis*, the sampling focused mainly on *Scrobipalpula* Povolný, 1964, although it also included the genera *Eurysacca* Povolný, 1967, *Gnorimoschema* Busck, 1900, *Magnifacia* Povolný, 1967, *Phthorimaea* Meyrick, 1902, *Scrobipalpulopsis* Povolný, 1987, and *Symmetrischema* Povolný, 1967. *Eurysacca* and *Magnifacia* were originally described as subgenera of *Scrobipalpula* (Povolný 1967). *Scrobipalpulopsis* was previously considered a synonym of *Scrobipalpula* (Lee et al. 2009). Some members of *Symmetrischema* have scale tufts on their hindwings (Povolný 1989), similar to those found in the new species. *Phthorimaea* is the genus in which *S. trichinaspis* was originally described (Meyrick 1917). *Gnorimoschema* is the type genus of Gnorimoschemini. *Eurysacca*, *Gnorimoschema*, *Scrobipalpula*, *Scrobipalpulopsis*, and *Symmetrischema* were represented by multiple species in BOLD, allowing at least two species of each to be included in the alignment. Since *Magnifacia* was represented only by its type species in BOLD, close matches were searched in this database, and the same procedure was followed for the new species. A single sequence of *Gelechia* Hübner [1825] was included to root the tree, as Gelechiini is the sister tribe of Gnorimoschemini (Karsholt et al. 2013). Genetic distances (p-distance) were estimated under the Kimura 2-parameter model (K2P) using MEGA11 (Tamura et al. 2021). Prior to phylogenetic reconstruction, the best-fitting nucleotide substitution model was identified in the ATGC bioinformatic platform (Guindon et al. 2010) using the Bayesian Information Criterion (BIC) implemented in Smart Model Selection (Lefort et al. 2017). The selected model, GTR+I+G, was subsequently applied in both ML and BI analyses. ML analyses were performed on the ATGC platform with node support estimated through 1000 bootstrap replicates. Bayesian analyses were conducted in MrBayes v. 3.2 (Huelsenbeck and Ronquist 2001) using two independent Markov Chain Monte Carlo runs of four chains each for 100 million generations, sampling every 10,000 generations. Convergence was assessed by verifying that effective sample sizes (ESS) exceeded 200, potential scale reduction factors (PSRF) approached 1.00, and an average standard deviation of split frequencies was below 0.01. After discarding 25% of samples as burn-in, a majority-rule consensus tree was computed under the half-compatibility criterion, with *Gelechia* designated as the outgroup. Bayesian analyses showed excellent convergence, with high ESS values and PSRF values approaching 1.0 for all parameters. Node support was assessed using bootstrap percentages (BP) in ML analysis and posterior probabilities (PP) in the BI analysis. Nodes with posterior probabilities ≥ 0.95 were considered strongly supported, whereas values between 0.90 and 0.95 were regarded as moderately supported. Bootstrap values ≥ 70% were considered indicative of supported clades (Hillis and Bull 1993; Lemoine et al. 2018). The resulting tree was visualised and edited in FigTree v. 1.4.4 (Rambaut 2018).

**Table 1.** DNA sequences of Gelechiidae used in the molecular analysis (bold indicates sequences generated in the present study).

**Tribe/Species**	**BOLD accession**
Gelechiini	
*Gelechia rhombella* ([Denis & Schiffermüller], 1775)	ABOLA335-14
Gnorimoschemini	
***Azaptilia azapensis* Vargas, gen. nov. et sp. nov**.	**NCMIC020-25**
***Azaptilia azapensis* Vargas, gen. nov. et sp. nov**.	**NCMIC021-25**
*Eurysacca melanocampta* (Meyrick, 1917)	LNAUU4648-15
*Eurysacca quinoae* Povolný, 1997	SENTO1281-18
*Gnorimoschema albimarginella* (Chambers, 1875)	LBCG081-08
*Gnorimoschema gallaesolidaginis* (Riley, 1869)	MNAK245-10
*Magnifacia aulorrhoa* (Meyrick, 1935)	NCNGS024-17
*Phthorimaea operculella* (Zeller, 1873)	ANICX1239-11
*Scrobipalpula artemisiella* (Kearfott, 1903)	MNAP032-12
*Scrobipalpula diffluella* (Frey, 1870)	LEAST925-17
*Scrobipalpula gutierreziae* Povolný & Powell, 2001	MNAJ362-09
*Scrobipalpula henshawiella* (Busck, 1903)	MNAK929-14
*Scrobipalpula manierreorum* Priest, 2014	ALLEP078-13
*Scrobipalpula psilella* (Herrich-Schäffer, 1854)	ABOLA839-15
*Scrobipalpula radiatella* (Busck, 1904)	BCMIN9507-23
*Scrobipalpula ramosella* (Müller-Rutz, 1934)	JSAM109-23
*Scrobipalpula sacculicola* (Braun, 1925)	LEPNF719-14
*Scrobipalpula tussilaginis* (Stainton, 1867)	CGUKD141-09
*Scrobipalpula wilsoni* Vargas, 2019	GBMNB43086-20
*Scrobipalpulopsis aguilaensis* Vargas, 2022	GBCAB33674-24
*Scrobipalpulopsis stirodes* (Meyrick, 1931)	NCNGS022-17
*Symmetrischema costaricanum* Povolný, 1990	BBLOD1969-11
*Symmetrischema tangolias* (Gyen, 1913)	GBMIN80431-17
unidentified gelechiid 1	GMAFD025-15
unidentified gelechiid 2	GMAFH108-15
unidentified gelechiid 3	GMAFN555-15

## Results

### Phylogenetic evidence

**Figure 1.**
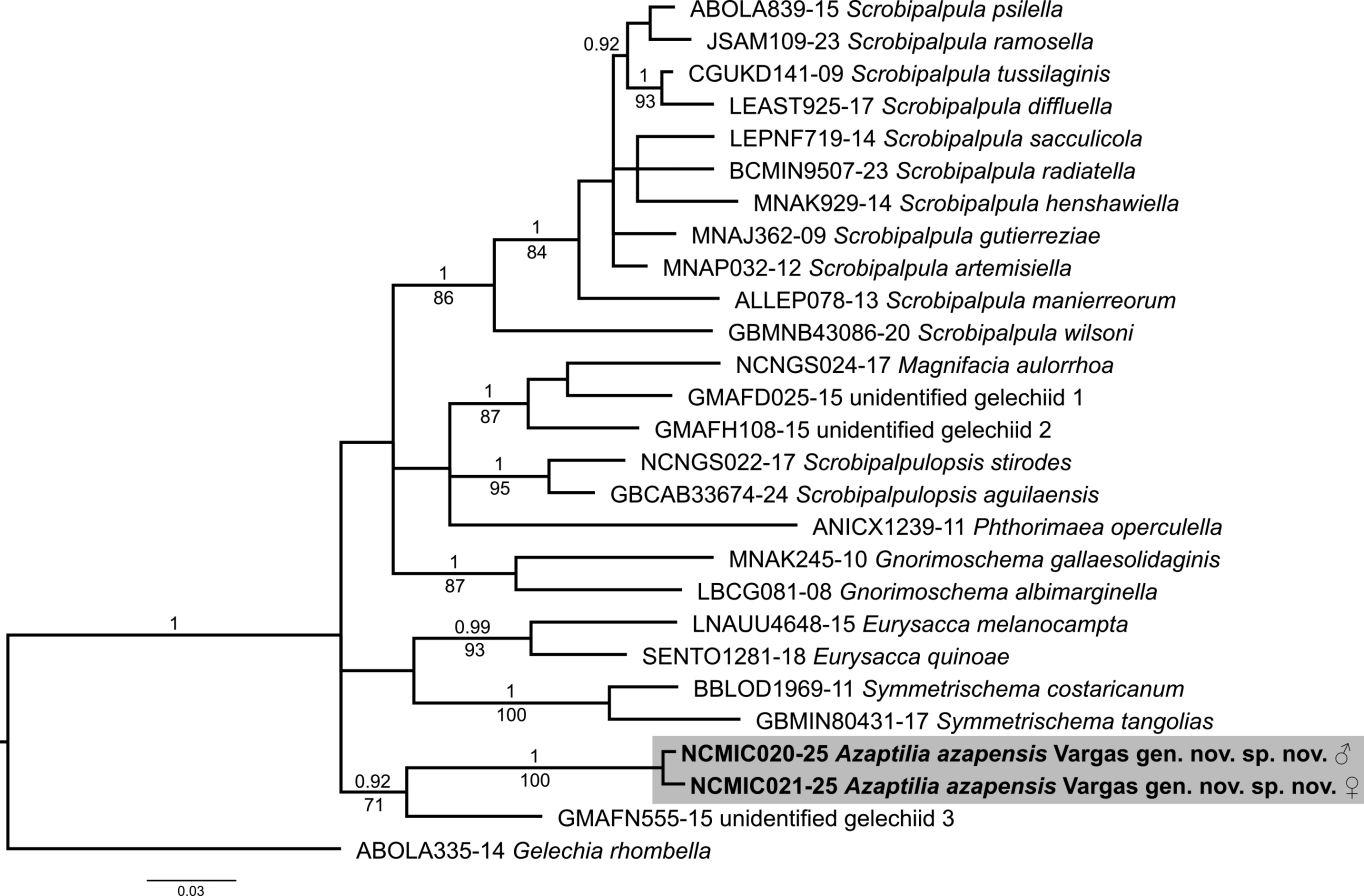
Bayesian majority-rule consensus tree of *Azaptilia azapensis* Vargas, gen. nov. et sp. nov. (bold) and representatives of Gnorimoschemini based on mitochondrial DNA sequences. The tree was rooted on *Gelechia* Hübner, [1825], a member of Gelechiini. Numbers above branches indicate node support (posterior probability) values ≥ 0.90. Numbers below branches indicate bootstrap support values ≥ 70 obtained in the maximum likelihood analysis.

The phylogenetic analysis (Fig. 1) provided strong support for the monophyly of all genera represented by multiple sequences and revealed that the two sequences of the new species form a well-supported clade, clearly separate from the other genera of Gnorimoschemini, sister to “unidentified gelechiid 3” from Formosa, Argentina (BOLD Process ID GMAFN555-15). This cluster received moderate support (PP = 0.92; BP = 71), exceeding commonly accepted bootstrap thresholds and falling within the range of moderate Bayesian support. The genetic divergence between the two sequences of the new species is 0.6% (K2P), while it is 7.1–7.2% between these and that of “unidentified gelechiid 3” (Table 2). Among the genera represented by multiple species, interspecific genetic divergence is 1.5–8.3% for *Scrobipalpula*, 2.7% for *Scrobipalpulopsis*, 3.9% for *Symmetrischema*, 4.9 for *Eurysacca*, and 7.1% for *Gnorimoschema*. The single representative of *Magnifacia* forms a well-supported clade with two unidentified gelechiids from Formosa, Argentina (BOLD Process IDs GMAFD025-15 and GMAFH108-15), with a genetic divergence of 4.9–5.5% between them.

**Table 2.** Genetic distances (K2P) among *Azaptilia azapensis* Vargas, gen. nov. et sp. nov. (bold) and representatives of Gnorimoschemini based on mitochondrial DNA sequences.

		**1**	**2**	3	4	5	6	7	8	9	10	11	12	13	14	15	16	17	18	19	20	21	22	23	24	25
**1**	***Azaptilia azapensis* Vargas, gen. nov. et sp. nov**.																									
**2**	***Azaptilia azapensis* Vargas, gen. nov. et sp. nov**.	**0.6**																								
3	*Eurysacca melanocampta*	**11.0**	**11.2**																							
4	*Eurysacca quinoae*	**9.5**	**9.3**	4.9																						
5	*Gnorimoschema albimarginella*	**9.8**	**10.0**	8.9	8.9																					
6	*Gnorimoschema gallaesolidaginis*	**10.3**	**10.5**	10.5	10.8	7.1																				
7	*Magnifacia aulorrhoa*	**8.1**	**8.2**	9.8	8.7	9.1	9.6																			
8	*Phthorimaea operculella*	**10.3**	**10.5**	10.0	8.4	9.9	11.0	8.9																		
9	*Scrobipalpula artemisiella*	**10.2**	**10.1**	10.1	9.4	9.4	10.3	10.3	11.0																	
10	*Scrobipalpula diffluella*	**11.4**	**11.4**	11.2	10.1	10.2	11.2	10.7	11.0	3.1																
11	*Scrobipalpula gutierreziae*	**10.5**	**10.5**	10.8	10.0	10.0	10.1	10.7	10.7	3.0	4.0															
12	*Scrobipalpula henshawiella*	**9.6**	**9.6**	10.5	9.3	9.1	9.8	9.6	10.0	3.8	4.9	4.3														
13	*Scrobipalpula manierreorum*	**10.5**	**10.7**	10.7	10.3	9.8	9.8	10.5	11.2	5.4	6.1	4.9	4.9													
14	*Scrobipalpula psilella*	**9.4**	**9.4**	10.3	8.6	9.1	10.3	10.0	10.0	2.5	2.5	3.0	3.9	5.1												
15	*Scrobipalpula radiatella*	**10.5**	**10.9**	10.0	9.1	9.6	11.0	10.1	11.2	3.3	4.1	4.3	3.6	5.1	3.8											
16	*Scrobipalpula ramosella*	**10.3**	**10.3**	11.0	9.6	9.6	10.1	11.0	10.7	2.5	3.1	3.3	4.3	5.4	1.5	3.8										
17	*Scrobipalpula sacculicola*	**9.6**	**10.0**	10.7	9.8	9.6	10.7	10.1	11.0	3.3	4.6	3.5	3.6	5.1	3.6	3.5	4.0									
18	*Scrobipalpula tussilaginis*	**10.3**	**10.3**	10.7	9.3	9.3	10.3	10.1	10.5	2.0	1.4	2.8	4.1	5.3	2.0	3.6	2.7	3.8								
19	*Scrobipalpula wilsoni*	**11.6**	**11.8**	11.0	9.8	10.5	11.6	10.0	11.4	7.3	7.9	7.8	7.6	8.3	7.8	7.7	7.6	7.4	7.4							
20	*Scrobipalpulopsis aguilaensis*	**8.7**	**8.9**	7.9	7.7	8.4	8.2	6.5	8.6	8.7	8.7	9.1	8.7	9.1	8.4	9.3	9.4	8.6	8.2	8.2						
21	*Scrobipalpulopsis stirodes*	**9.1**	**9.3**	7.9	7.5	8.6	7.9	5.9	8.2	9.6	9.9	9.6	8.7	10.0	9.2	9.4	10.3	9.1	9.4	8.9	2.7					
22	*Symmetrischema costaricanum*	**9.8**	**10.0**	8.9	7.9	8.7	11.5	10.1	10.5	10.1	10.7	9.3	9.6	9.6	8.9	10.1	9.6	9.2	9.8	9.9	9.2	9.8				
23	*Symmetrischema tangolias*	**10.3**	**10.5**	9.3	8.4	9.3	11.5	11.3	11.3	10.6	11.5	10.5	10.5	10.5	10.5	10.6	10.6	10.6	10.6	10.7	10.6	11.3	3.9			
24	unidentified gelechiid 1	**8.4**	**8.6**	8.7	8.0	9.3	9.4	4.9	9.4	7.9	9.3	9.1	9.3	9.6	8.6	9.6	9.6	8.8	8.7	9.1	6.2	6.0	9.2	10.6		
25	unidentified gelechiid 2	**9.3**	**9.5**	9.6	8.4	9.8	10.1	5.5	10.1	8.2	8.8	9.1	8.4	9.1	8.1	8.1	8.8	8.6	8.2	8.6	6.4	6.4	9.8	10.3	4.9	
26	unidentified gelechiid 3	**7.1**	**7.2**	9.2	8.1	8.9	9.8	7.9	9.3	9.3	9.8	9.1	8.7	9.1	7.9	9.3	9.3	8.2	9.3	9.1	6.7	6.4	8.2	9.1	7.7	8.2

### Taxonomy

#### Azaptilia

Taxon classificationAnimaliaLepidopteraGelechiidae

Vargas gen. nov.

733459C2-B2B0-59F7-9F7F-1FDA48FA5F28

https://zoobank.org/13787E45-8C89-4709-8430-8F10B07BDF7D

##### Type species.

*Azaptilia azapensis* Vargas, sp. nov., designed here.

##### Etymology.

The genus name is derived from the name of the type locality (Azapa Valley) of the type species and “-ptilia”, a Latinization of the Greek word “πτίλον” (plumage). *Azaptilia* is considered feminine in gender.

##### Diagnosis.

*Azaptilia* is recognized by the following combination of morphological characters: 1) scale tuft on male hindwing, 2) uncus with a triangular postero-medial projection, and 3) U-shaped gnathos. Among the South American Gnorimoschemini, the U-shaped gnathos of *Azaptilia* resembles that of members of *Phthorimaea*, such as *Phthorimaea perfidiosa* Meyrick, 1917 from Colombia (Povolný 1967: fig. 103) and *Phthorimaea euchthonia* Meyrick, 1939 from Argentina (Povolný 1967: fig. 104), and some representatives of *Scrobipalpula*, such as *Scrobipalpula acuta* Povolný, 1990 from Peru (Povolný 1990: fig. 47). However, these three species lack a scale tuft on their male hindwings. Furthermore, the triangular postero-medial projection of the uncus of *Azaptilia* contrasts with the truncate uncus in *P. perfidiosa* and *P. euchthonia*, and the rounded tip of the uncus in *S. acuta*. The scale tuft on the male hindwing of *Azaptilia* resembles that described for *Symmetrischema nanum* Povolný, 1989 and *Symmetrischema oblitum* Povolný, 1989 from Argentina (Povolný 1989). However, the U-shaped gnathos of *Azaptilia* contrasts with the narrow, short gnathos of these two species. The triangular postero-medial projection of the uncus of *Azaptilia* resembles the uncus of *Magnifacia aulorrhoa* (Meyrick, 1935) from southern Argentina (Povolný 1987: figs 73–75). However, the male of *M. aulorrhoa* lacks a tuft of scales on the hindwing, and has a Y-shaped gnathos in the genitalia.

##### Species included.

*Azaptilia* includes the following two species:

*Azaptilia azapensis* Vargas, sp. nov.

*Azaptilia trichinaspis* (Meyrick, 1917), comb. nov.

##### Distribution.

*Azaptilia* is known from the arid environments of the Atacama (Chile) and Sechura (Peru) deserts.

#### Azaptilia azapensis

Taxon classificationAnimaliaLepidopteraGelechiidae

Vargas sp. nov.

CB328CC9-69F6-5E1E-8B32-B89053E9DD36

https://zoobank.org/2E6E8F92-E26D-454C-9219-88D852A07608

**Figure 2.**
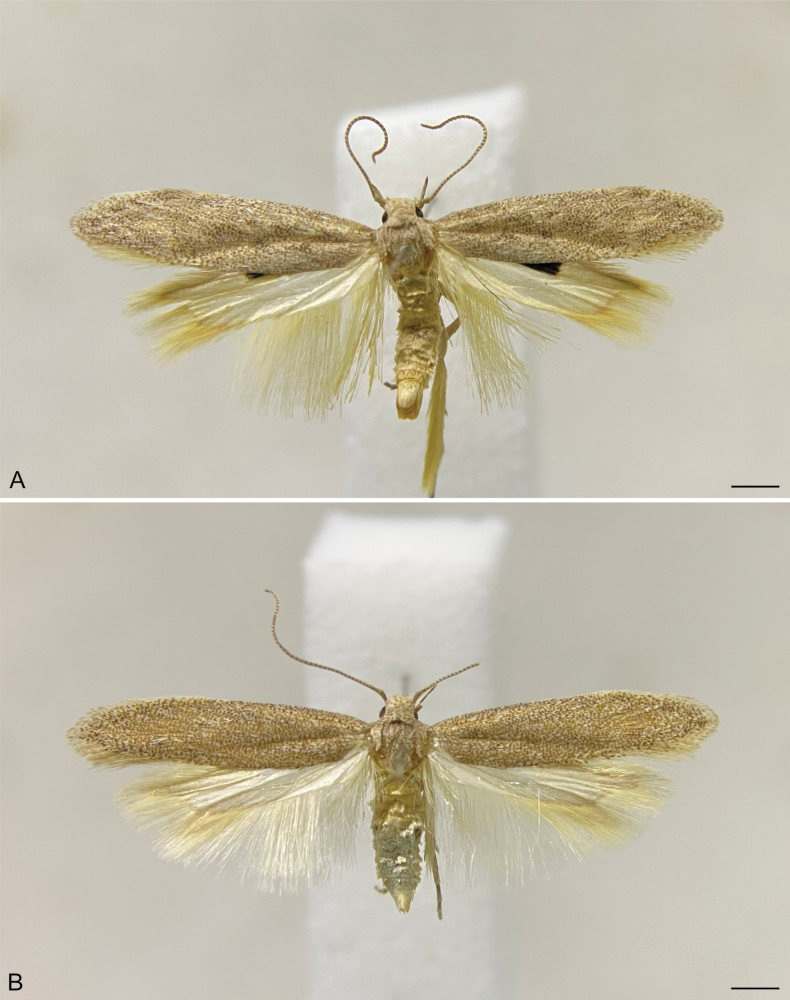
Habitus of *Azaptilia azapensis* Vargas, gen. nov. et sp. nov. **A**. Holotype, male, dorsal; **B**. Paratype, female, dorsal. Scale bars: 1 mm (**A, B**).

**Figure 3.**
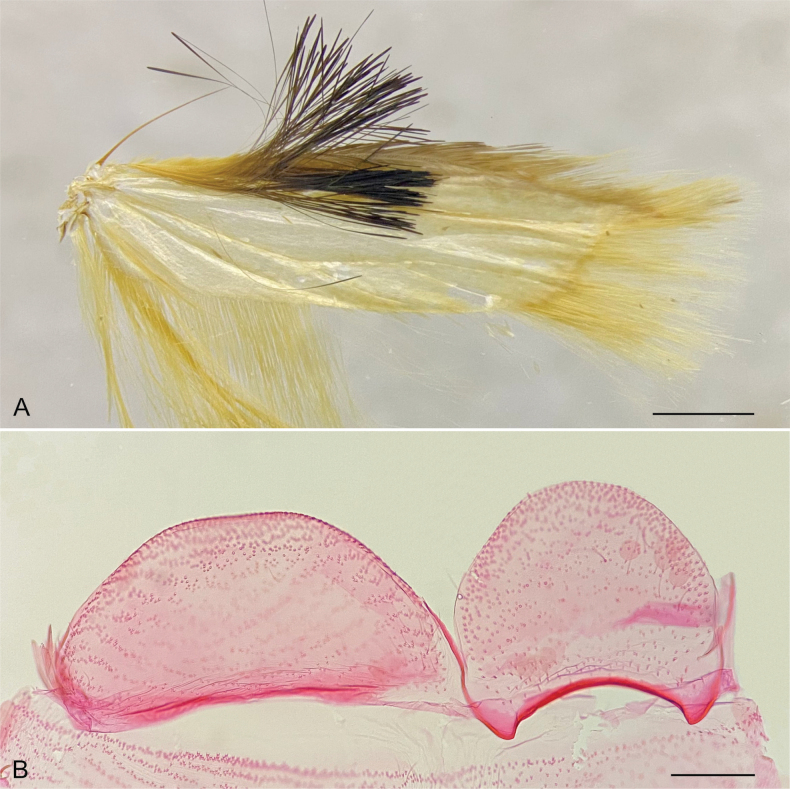
Sexually dimorphic structures of the male of *Azaptilia azapensis* Vargas, gen. nov. et sp. nov. **A**. Hindwing with scale tuft; **B**. Abdominal segment VIII, sternum to the left, tergum to the right. Scale bars: 1 mm (**A**); 0.2 mm (**B**).

**Figure 4.**
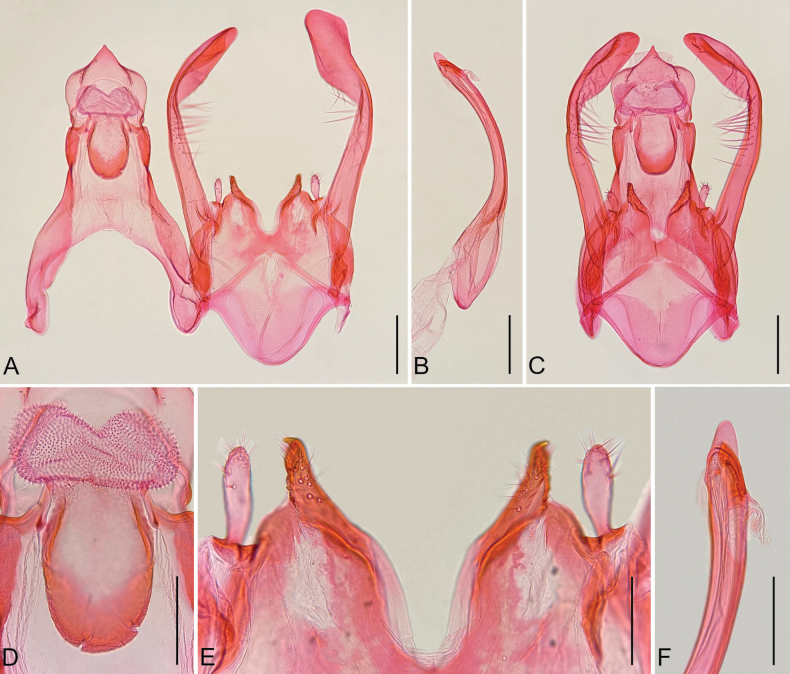
Male genitalia of *Azaptilia azapensis* Vargas, gen. nov. et sp. nov. **A**. Unrolled, phallus removed, left part ventral, right part dorsal; **B**. Phallus, lateral; **C**. Ventral view, phallus removed; **D**. Gnathos and culticulla; **E**. Vincular processes (central) and sacculus (lateral); **F**. Apex of the phallus. Scale bars: 0.2 mm (**A–C**); 0.1 mm (**D–F**).

**Figure 5.**
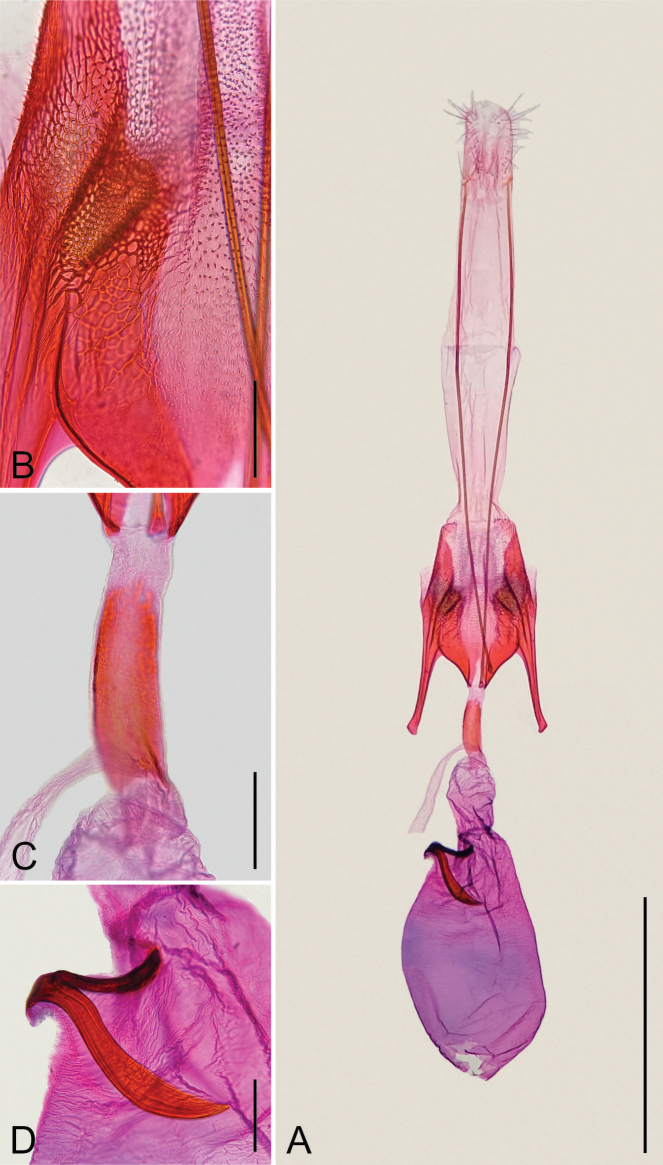
Female genitalia of *Azaptilia azapensis* Vargas, gen. nov. et sp. nov. **A**. Ventral view; **B**. Detail of the antrum; **C**. Colliculum; **D**. Signum. Scale bars: 1 mm (**A**); 0.1 mm (**B–D**).

**Figure 6.**
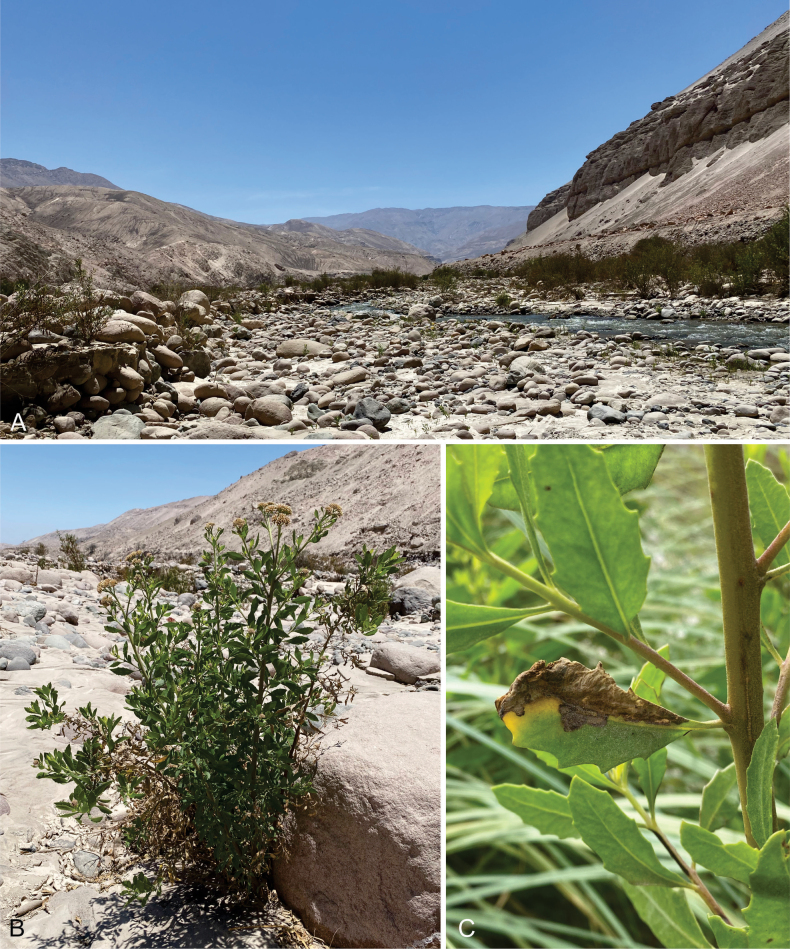
Natural history of *Azaptilia azapensis* Vargas, gen. nov. et sp. nov. **A**. Type locality, Azapa Valley, Arica Province, northern Chile; **B**. Host plant, *Tessaria absinthioides* (Hook. & Arn.) DC. (Asteraceae), at the type locality; **C**. Typical aspect of a leaf of *T. absinthioides* mined by a larva of *A. azapensis*.

**Figure 7.**
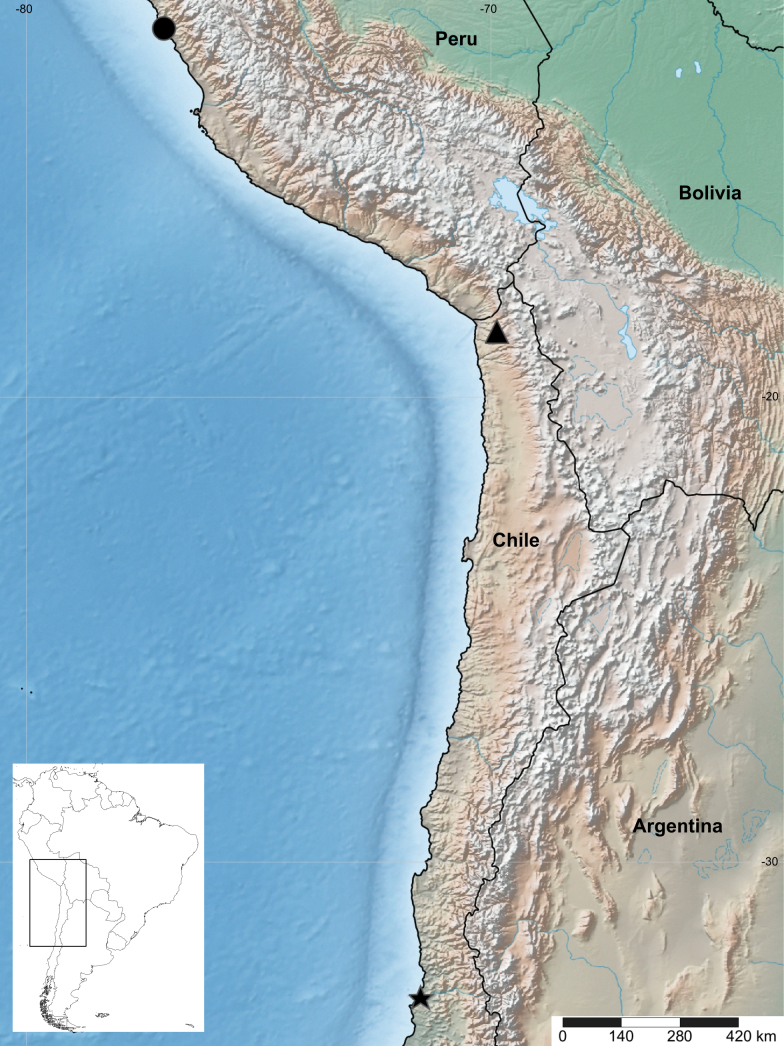
Distribution records of the two species of *Azaptilia* Vargas, gen. nov. Circle indicates the type locality of *Azaptilia trichinaspis* (Meyrick, 1917) from the Sechura Desert, Peru. Triangle indicates the type locality of *Azaptilia azapensis* sp. nov. from the Atacama Desert, Chile. Star indicates the sampling site of an unidentified leaf miner gelechiid associated with *Tessaria absinthioides* (Hook. & Arn.) DC. (Asteraceae) in Central Chile.

Figs 1, 2, 3, 4, 5, 6, 7

##### Type locality.

Azapa Valley (18°35'05"S, 69°52'19"W), at about 1050 m elevation in the Atacama Desert, Arica Province, northern Chile.

##### Type material.

***Holotype***. Chile • ♂; Azapa, Arica, December 2024; H.A. Vargas leg.; ex-larva; *Tessaria absinthioides*; October 2024; “HOLOTYPE *Azaptilia azapensis* Vargas” [red handwritten label]; IDEA-LEPI-2025-09; HAV-1876 [genitalia slide]; BOLD Process ID NCMIC020-25 (IDEA). ***Paratypes***. Chile • 3♂ 3♀, same data as for the holotype IDEA-LEPI-2025-10 to IDEA-LEPI-2025-15; HAV-1877, 1878, 1879, 1884, 1885, 1890 [genitalia slides]; BOLD Process ID NCMIC021-25 (IDEA).

##### Diagnosis.

*Azaptilia azapensis* is recognized by the presence of a tuft of scales arising near the base of the costa on the upper surface of the male hindwings (Figs 2A, 3A); the relatively short vincular processes not overlapping the gnathos, and the suddenly narrowed tip of the vincular processes in the male genitalia (Fig. 4); and the elongated colliculum, about three times longer than wide, in the female genitalia (Fig. 5). The genitalia of *A. azapensis* strongly resemble those of the only other congeneric *A. trichinaspis*. However, the scale tuft arising posterior to the discal cell on the lower surface of the male hindwings (Meyrick 1917; Clarke 1969: plate 83, fig. 2); the longer, finger-like vincular processes overlapping the gnathos in the male genitalia (Povolný 1967: fig. 79); and the shorter, square-shaped colliculum in the female genitalia of *A. trichinaspis* (Povolný 1967: fig. 78) clearly contrast with *A. azapensis*.

##### Description.

**Male** (forewing length 6.1–6.2 mm) (Figs 2, 3). Head. Frons and labial palp having yellowish-white scales with greyish-brown tip; vertex, maxillary palp and haustellum yellowish white; antenna yellowish white, filiform, length about two-thirds the forewing. Thorax. Yellowish-white scales with greyish-brown tip dorsally; yellowish-white laterally; fore- and midleg with yellowish-white scales with greyish-brown tip; hindleg yellowish white; forewing with yellowish-white scales with greyish-brown tip; fringe greyish brown; hindwing yellowish white; a tuft of scales (Figs 2A, 3A) arising near base of costa on upper surface and extending to about 2/3, light yellowish brown at base, gradually darkening towards tip, becoming black in distal half; fringe yellowish-white. Abdomen. Yellowish white; tergum VIII tongue-like, length in middle about 0.8 times maximum width, anterior margin broadly concave and strongly sclerotized, posterior margin rounded; sternum VIII semicircular, about 0.9 times the length and 1.3 times the wide of tergum VIII (Fig. 3B). ***Male genitalia*** (Fig. 4): Tegumen Y-shaped with deep and broadly concave anterior notch. Uncus a short transverse plate with broadly concave anterior margin, and a triangular postero-medial projection. Gnathos U-shaped, slightly widened in middle. Culticula with abundant microtrichia. Vinculum with deep posterior cleft in middle separating vincular processes, almost reaching posterior margin of saccus; vincular processes with suddenly narrowed tip, not overlapping gnathos; narrowed part of vincular processes setose and with acute apex. Saccus diamond-shaped with rounded anterior tip, about 1.2 times wider than long; partially separated from vinculum by straight oblique cuticular thickenings. Valva mostly narrow and straight; slightly widened and inwardly curved tip; medial third with setose inner side. Sacculus finger-like, setose, short, almost reaching tip of vincular processes. Phallus narrow, elongated, curved, similar in length to valva, with small semicircular projection and small sclerite at tip; coecum swollen.

**Female** (Fig. 2B). Similar to male in size and coloration, but hindwing lacking tuft of scales, and abdominal segment VIII not modified as in male. ***Female genitalia*** (Fig. 5): Papillae anales broadly rounded posteriorly, with a few scattered setae. Posterior apophyses rod-shaped, narrow, length about 6.5 times the papillae anales, tip reaching near tip of antrum. Anterior apophyses rod-shaped, stout, length about 0.16 times the posterior apophyses. Tergum VIII interrupted mid-dorsally, latero-ventrally continuous with anterior apophyses and antrum. Antrum gradually narrowing forward; tip reaching about half the length of anterior apophysis; sclerotized lateral part irregularly sculptured; membranous medial part with microtrichia. Ductus bursae with short membranous posterior part and long colliculum about half the length of antrum; inception of ductus seminalis near tip of colliculum. Corpus bursae pear-shaped, mostly membranous, length about 0.6 times the posterior apophyses, with a slightly curved sword-shaped signum with acute tip.

##### Etymology.

The species name is derived from the name of the type locality (Azapa Valley).

##### Distribution and natural history

**(Figs 6, 7)**. *Azaptilia azapensis* is a little-known moth. It has been collected only at the type locality, the Azapa Valley, in the Atacama Desert, northern Chile. Its larvae are leaf miners on *T. absinthioides*, a shrub occurring in Argentina, Bolivia, Chile, and Peru, whose Chilean range extends from 18 to 37°S, from sea level to approximately 3000 m altitude (Rodriguez et al. 2018).

#### Azaptilia trichinaspis

Taxon classificationAnimaliaLepidopteraGelechiidae

(Meyrick, 1917) comb. nov.

81CC838E-535F-51FD-A60D-6FE9C05FC1CC

Phthorimaea trichinaspis Meyrick, 1917, p. 167.
Scrobipalpula trichinaspis (Meyrick, 1917): Povolný (1967), p. 93; Becker (1984), p. 47; Hobern et al. (2025).
Gnorimoschema trichinaspis (Meyrick, 1917): Clarke (1969), 167.

##### Type locality.

Peru, Lima (Meyrick 1917).

##### Diagnosis.

See that for *A. azapensis*.

##### Distribution and natural history.

Only known from the type locality (Meyrick 1917) (Fig. 7). The type material was collected in August (Meyrick 1917).

## Discussion

The discovery of *A. azapensis* aligns with a broader biogeographic pattern of Lepidoptera diversification in the arid and hyper-arid environments of northern Chile. Coastal valleys and the Andean foothills of the Atacama Desert have increasingly been recognized as reservoirs of endemic and phylogenetically distinct lineages, including the geometrid *Physocleora polyphaga* Vargas-Ortiz & Parra, 2025 from riparian and montane habitats (Vargas-Ortiz and Parra 2025), and the gracillariid *Atacamaptilia ambrosiavora* Vargas & Espinoza-Donoso, 2022, a leaf miner associated with *Ambrosia cumanensis* Kunth (Asteraceae) (Espinoza-Donoso et al. 2022). Additional cases, such as the sphingid *Hyles annei* (Guérin-Méneville, 1839), which relies on ephemeral host plants that appear after sporadic rainfall, further illustrate how extreme climatic variability shapes Lepidoptera persistence and diversification in this region (Vargas and Hundsdoerfer 2019). Collectively, these examples indicate that the Atacama functions not merely as an ecological barrier but also as an evolutionary cradle in which habitat specialization, host-plant fidelity, and microhabitat isolation promote lineage divergence. Phylogeographic evidence from other Atacama species supports this interpretation, as studies on coastal insects show complex patterns of colonization over time and space as well as long-term isolation, which are consistent with the existence of desert micro-refuges (Campos-Soto et al. 2022). Similar patterns have been reported in tenebrionid beetles of the Puna-Atacama Province, where sister clades exhibit non-overlapping distributions shaped by historical isolation and habitat specialization (Zúñiga-Reinoso et al. 2021). In this context, the deep COI divergence and unique genitalia morphology of *Azaptilia* support the hypothesis of long-term persistence within isolated desert habitats. These findings emphasize the need for continued systematic sampling across Atacama valleys to elucidate the evolutionary history of Neotropical Gnorimoschemini and deepen our understanding of diversification processes in one of the world’s most extreme ecosystems.

The geographic range of *T. absinthioides* extends beyond the southern boundary of the Atacama Desert, reaching approximately 37°S in southern Chile (Rodriguez et al. 2018). Surprisingly, field observations by the first author of the present study in central Chile, almost 1600 km south of the Azapa Valley, revealed leaf mines on *T. absinthioides* like those built by *A. azapensis*. Based on the above-mentioned examples dealing with other insect taxa endemic to the hyper-arid environments of the western margin of South America (Zúñiga-Reinoso et al. 2021; Campos-Soto et al. 2022), further studies are encouraged to assess whether additional species of *Azaptilia* have differentiated along the broad latitudinal gradient inhabited by *T. absinthioides*, or whether other unrelated lepidopteran lineages have assumed the role of leaf miners on this shrub outside the Atacama Desert.

The recognition of *A. trichinaspis* as a member of *Azaptilia* highlights the historical relationships between the contiguous Atacama and Sechura deserts (Porter 1985; Howden 2008). Among the lepidopteran species inhabiting this area, some with high dispersal capacity can maintain gene flow between their geographically isolated populations, such as the nymphalid butterfly *Dione dodona* Lamas & Farfán, 2022, whose geographic range encompasses much of these two deserts (Farfán et al. 2022). In contrast, pairs of putative closely related species with very restricted allopatric distributions have been reported for some moth genera (Clarke 1987; Espinoza-Donoso et al. 2020). In addition, a recent DNA barcoding analysis revealed that the leaf miner gracillariid genus *Angelabella* Vargas & Parra, 2005, originally described to include only the type species from the Atacama Desert, likely harbours three additional cryptic allopatric species in the Sechura Desert (Vargas-Ortiz et al. 2020). Therefore, further surveys across the Sechura Desert would be valuable for better characterizing the geographic distribution of *A. trichinaspis* and assessing the potential presence of additional members of the *Azaptilia*.

The establishment of *Azaptilia* expands the known generic diversity of Gnorimoschemini and highlights the Atacama and Sechura deserts as important reservoirs of Lepidoptera biodiversity, emphasizing the value of integrative taxonomy for documenting and understanding biodiversity in extreme arid ecosystems.

## Supplementary Material

XML Treatment for Azaptilia

XML Treatment for Azaptilia azapensis

XML Treatment for Azaptilia trichinaspis

## Additional information

### Artificial Intelligence (AI) use

The authors accept full responsibility for the content of the manuscript, including the disclosure of any use of AI.

No AI tools were used in the preparation of this manuscript.

### Author ORCIDs

J. Ampuero-Vega https://orcid.org/0000-0002-3605-7039

R. Campos-Soto https://orcid.org/0000-0001-7052-0501

H.A. Vargas https://orcid.org/0000-0002-5355-3157

### Data availability

All of the data that support the findings of this study are available in the main text.
